# A simple score of sarcopenic obesity predicts all-cause mortality in patients with cirrhosis

**DOI:** 10.3389/fmed.2026.1719899

**Published:** 2026-01-22

**Authors:** Yuteng Yang, Tingyang Wei, Shuo Yang, Ran Fei, Changjie Tie, Rui Han, Danli Ma, Qian Jin, Jian Wang, Zixing Wang, Rui Huang

**Affiliations:** 1 Peking University People’s Hospital, Beijing, China; 2 School of Basic Medical Sciences, Peking University Health Science Center, Beijing, China; 3 Department of Radiology, Peking University People’s Hospital, Beijing, China; 4 Beijing Key Laboratory of Hepatitis C and Immunotherapy for Liver Diseases, Beijing International Cooperation Base for Science and Technology on NAFLD Diagnosis, Infectious Disease and Hepatology Center of Peking University People's Hospital, Peking University Hepatology Institute, Peking University People's Hospital, Beijing, China; 5 Department of Internal Medicine, Harbin Pingfang District People's Hospital, Harbin, China

**Keywords:** body composition, computed tomography, liver cirrhosis, prognosis, sarcopenic obesity

## Abstract

**Purpose:**

To evaluate the predictors and prognostic impact of sarcopenic obesity (Sa-O) on all-cause mortality in patients with cirrhosis.

**Methods:**

This retrospective cohort study included cirrhosis patients. Sa-O was defined using computed tomography at L3 level as a skeletal muscle index <42 cm^2^/m^2^ or <38 cm^2^/m^2^ in men and women, respectively, with a visceral adipose tissue area >100 cm^2^. The primary outcome was all-cause mortality. A nomograph was developed based on identified predictors, generating the age–body mass index (BMI)–alcohol–hypertension (ABAH) score.

**Results:**

Among 769 cirrhosis patients (60.1% male; 45.5% aged ≥60 years; median follow-up: 4.3 [1.7–7.0] years), 129 (16.8%) were diagnosed with Sa-O. Multivariable analysis identified age (OR [odds ratio] 1.813, 95% confidence interval [CI] 1.210–2.718, *p* = 0.0040), BMI ≥ 28 kg/m^2^ (OR 0.076, 95% CI 0.018–0.317, *p* = 0.0011), alcoholic liver disease (OR 1.685, 95% CI 1.078–2.634, *p* = 0.0220), and hypertension (OR 1.801, 95% CI 1.184–2.739, *p* = 0.0059) as independent predictors of Sa-O. As for BMI (OR 0.076), the limitations of BMI in reflecting body composition led to the counterintuitive research findings. When stratified by ABAH score into low (<100), medium (100–129), and high (≥130) score groups, patients demonstrated progressively higher rates of Sa-O (8.4, 18.7, and 30.5%, respectively; *p* = 0.026). The prognostic value of the ABAH score was analyzed using a Cox proportional hazards model by subgroups analysis. It showed significant prognostic value for all-cause mortality, with medium-score patients exhibiting a 1.53-fold increased risk (95% CI 1.12–2.09, *p* = 0.007) and high-score patients a 1.72-fold increased risk (95% CI 1.22–2.42, *p* = 0.002) compared to the low-score reference group.

**Conclusion:**

Sa-O in patients with cirrhosis is associated with age, BMI, alcoholic liver disease, and hypertension. The newly developed ABAH score, a composite clinical risk marker, predicted the probability of Sa-O and was associated with all-cause mortality in patients with cirrhosis.

## 1 Introduction

Cirrhosis is a progressive complication of most chronic liver conditions and imposes a significant global burden on health. In 2017, it affected 0.3–0.8% of the global population (1) and accounted for 2.4% of all deaths in 2019, ranking as the 11th leading cause of death worldwide (2, 3). Cirrhosis often leads to sarcopenia. Sarcopenia prevalence in patients with cirrhosis ranges from 40 to 70% (4). Obesity exacerbates cirrhosis progression, given its rising global prevalence. Although insulin resistance contributes to sarcopenic obesity—defined as the coexistence of sarcopenia and obesity—which is distinct from either condition alone and often portends a worse prognosis—patients with cirrhosis develop this condition primarily through distinct mechanisms, including impaired protein synthesis, chronic inflammation, and metabolic dysfunction (5–7). These individuals often present with portal hypertension and gut dysbiosis, leading to poorer clinical outcomes than in non-cirrhotic populations (7). Although sarcopenic obesity in cirrhosis has been reported in 2–42% of cases and linked to decreased survival and poor prognosis, the current evidence remains limited (7–9). Therefore, early identification of sarcopenic obesity is crucial for individualized treatment of cirrhosis.

Most diagnostic criteria for sarcopenic obesity rely on computed tomography (CT) imaging at the L3 vertebral level to assess skeletal muscle mass and visceral adipose tissue area (VATA) (10, 11). For patients with cirrhosis, CT is a practical diagnostic tool, as it is routinely used in abdominal follow-up imaging and enables accurate quantification and differentiation of fat compartments. Therefore, CT-derived body composition parameters may offer diagnostic value for sarcopenic obesity. Previous studies have linked sarcopenic obesity in cirrhosis patients to higher mortality, shorter median survival, and increased susceptibility to infection-related deaths (8). However, the diagnostic criteria for sarcopenic obesity have not been validated in cirrhotic populations, and studies predominantly comprising end-stage liver disease are limited (8). Therefore, large-scale studies on the incidence and contributing factors of sarcopenic obesity in cirrhosis remain limited, and its predictive value remains unclear. Moreover, there is a lack of simple, clinically accessible predictive models or scores for sarcopenic obesity in cirrhosis, highlighting the need for a new tool.

To this end, in this study, we aimed to assess the predictors of sarcopenic obesity and develop a predictive model for this condition in patients with cirrhosis. We also explored the predictive value of sarcopenic obesity relative to all-cause mortality in this population. Clinically, the findings may help optimize stratified management and inform targeted interventions for patients with cirrhosis.

## 2 Methods

### 2.1 Study design and participants

This retrospective study included patients diagnosed with cirrhosis between November, 2008 and September, 2022 at Peking University People’s Hospital. Eligible participants were adults (≥18 years) with comprehensive medical records, a CT scan, and a follow-up period exceeding 6 months after baseline imaging. The exclusion criteria were as follows: (i) liver transplantation, (ii) hepatocellular carcinoma, (iii) other neoplasms, and (iv) chronic kidney disease or nephrotic syndrome. This study was approved by the Ethics Committee of Peking University People’s Hospital (approval no. 2023PHB214-001). Given the retrospective nature of the research, the need for informed consent was waived by the committee.

Clinical data collected for each patient included age, sex, body mass index (BMI), etiology of liver disease (viral, alcoholic, others, multiple causes), comorbidities (hypertension, diabetes), complications (ascites, spontaneous bacterial peritonitis, variceal bleeding, and hepatic encephalopathy), decompensated cirrhosis, and follow-up duration. All disease information was collected according to the International Classification of Diseases (12).

### 2.2 Body composition based on CT images

Body composition was assessed using CT scans at L3 level, measuring skeletal muscle area (SMA) and VATA. SMA was normalized to height squared (m^2^) to derive the skeletal muscle index (SMI; cm^2^/m^2^). Given the lack of academic consensus on the diagnostic criteria for sarcopenic obesity in patients with cirrhosis, sarcopenic obesity was defined as the coexistence of sarcopenia and obesity (13). Sarcopenia was diagnosed in patients with end-stage liver disease as SMI < 38 cm^2^/m^2^ in females and <42 cm^2^/m^2^ in males, according to the Japan Society of Hepatology, whereas obesity was diagnosed as VATA >100 cm^2^, as recommended by the Japan Society for the Study of Obesity (11). These criteria were chosen because they are well-established for Asian populations and are routinely used in clinical practice in our setting, but they may have limitations in other ethnicities.

### 2.3 Study outcome

Based on the duration of follow-up from the initial admission, the primary outcome was set as all-cause mortality as of August 31, 2023.

### 2.4 Statistical analysis

Continuous variables are presented as medians with interquartile ranges, whereas categorical data are summarized using percentages. Patients with cirrhosis were classified into two groups: with and without sarcopenic obesity. Group differences were assessed using Wilcoxon rank-sum and chi-square tests, as appropriate. Univariate and multivariate analyses were performed to identify sarcopenic obesity predictors. Variables with *p* < 0.2 in univariate analysis were included in multivariate analysis, and stepwise regression was used to identify significant predictors at *p* < 0.05. Nomographs were developed to integrate age, BMI, alcohol consumption, and hypertension, which were identified as predictors of sarcopenic obesity in multivariate analysis. The points for each variable—age (≥60 years), BMI (normal <24 kg/m^2^, overweight 24–28 kg/m^2^, obese ≥28 kg/m^2^), alcohol-related cirrhosis, and comorbid hypertension—were directly derived from the beta coefficients of the final multivariate logistic regression model. The scoring system was created by proportionally assigning points to each variable based on the relative weight of its coefficient, following established methods for converting a regression model into a points-based scoring system. Total points formed the age–BMI–alcohol–hypertension (ABAH) score, which was mapped to a linear predictor to estimate the probability of sarcopenic obesity. The risk categories (Low: <100, Medium: 100–129, High: ≥130) were defined post-hoc based on the distribution of the total ABAH score in our cohort. The cut-offs were determined by splitting the entire population into three groups of equal size (tertiles) for exploratory risk stratification, as no established clinical thresholds exist. The prognostic value of the ABAH score was analyzed using a Cox proportional hazards model by subgroups analysis. For this analysis, the ABAH score was incorporated as a three-category variable (Low, Medium, High), based on the tertiles described in the score development section. The resulting hazard ratios (HRs) were adjusted for age, sex, and MELD score. The MELD score was chosen for adjustment as it is a well-validated measure of overall liver disease severity. The significance level was set at *p* = 0.05. Basic analyses were conducted using SAS 9.4, whereas the competing risks model was implemented with the R package “cmprsk” (2.2.12).

## 3 Results

The final analysis comprised 769 patients, with a median follow-up duration of 4.3 (1.7–7.0) years.

### 3.1 Baseline characteristics

Among the included patients, 350 (45.5%) were aged >60 years, and 462 (60.1%) were male. A total of 416 (54.1%) patients had decompensated cirrhosis, and 129 (16.8%) were diagnosed with sarcopenic obesity. Compared with patients without sarcopenic obesity, those with sarcopenic obesity were older (≥60 years: 60.5% vs. 42.5%, *p* = 0.0002) and had lower BMI (BMI < 24 kg/m^2^: 69.8% vs. 52.7%, *p* < 0.0001). We also observed a rising prevalence of hypertension (39.5% vs. 25.6%, *p* = 0.0013) and diabetes (29.5% vs. 20.9%, *p* = 0.0341) in patients with sarcopenic obesity (Table 1).

**Table 1:** Baseline characteristics of patients with cirrhosis by sarcopenic obesity (*n* = 769).

Variables	Total	Sarcopenic obesity		
	(*n* = 769)	Yes (*n* = 129)	No (*n* = 640)	*P*
Age (years)				
<60	419	51 (39.5)	368 (57.5)	0.0002
≥60	350	78 (60.5)	272 (42.5)	
Gender, *n* (%)				
Male	462	69 (53.5)	393 (61.4)	0.0939
Female	307	60 (46.5)	247 (38.6)	
BMI (kg/m^2^), *n* (%)				<0.0001
<24	427	90 (69.8)	337 (52.7)	
24–28	243	37 (28.7)	206 (32.2)	
≥28	99	2 (1.6)	97 (15.2)	
Etiology of liver diseases, *n* (%)				0.0349
Viral	310	40 (31.0)	270 (42.2)	
Alcoholic	164	10 (7.8)	64 (10.0)	
Others	221	37 (28.7)	127 (19.8)	
Multiple causes	74	42 (32.6)	179 (28.0)	
Co-morbidities, *n* (%)				
Hypertension	215	51 (39.5)	164 (25.6)	0.0013
Diabetes	172	38 (29.5)	134 (20.9)	0.0341
Complications, *n* (%)				
Ascites	417	66 (51.2)	351 (54.8)	0.4439
Spontaneous bacterial peritonitis	85	19 (14.7)	66 (10.3)	0.1445
Variceal bleeding	36	9 (7.0)	27 (4.2)	0.1761
Hepatic encephalopathy	100	17 (13.2)	83 (13.0)	0.9485
Decompensated cirrhosis, *n* (%)	416	65 (50.4)	351 (54.8)	0.3542
MELD score	606	9.0 (3.0–11.6)	8.5 (4.5–11.8)	0.8293
Follow-up duration (years)	769	4.3 (1.7–6.8)	4.4 (1.7–7.1)	0.9097

Categorical values are shown as *n* (%). Continuous variables are shown as median (IQR). BMI, body mass index; MELD, model for end-stage liver disease.

### 3.2 Predictors of sarcopenic obesity in patients with cirrhosis

In the univariable analysis, age ≥60 years (reference: <60 years) was significantly associated with sarcopenic obesity in patients with cirrhosis (odds ratio [OR] 2.069, 95% confidence interval [CI] 1.406–3.045, *p* = 0.0002). BMI (reference: normal weight, <24 kg/m^2^) demonstrated reduced odds for both overweight (24–28 kg/m^2^: OR 0.077, 95% CI 0.019–0.319, *p* = 0.0011) and obese (≥28 kg/m^2^: OR 0.077, 95% CI 0.019–0.319, *p* = 0.0011) individuals compared to those with normal weight. Additional predictors included alcoholic liver disease (OR 1.967, 95% CI 1.200–3.224, *p* = 0.0300), hypertension (OR 1.898, 95% CI 1.278–2.817, *p* = 0.0015), and diabetes (OR 1.577, 95% CI 1.032–2.409, *p* = 0.0352). In the multivariable analysis, age ≥60 years (OR 1.813, 95% CI 1.210–2.718, *p* = 0.0040), obesity (BMI ≥ 28 kg/m^2^: OR 0.076, 95% CI 0.018–0.317, *p* = 0.0011), alcoholic liver disease (OR 1.685, 95% CI 1.078–2.634, *p* = 0.0220), and hypertension (OR 1.801, 95% CI 1.184–2.739, *p* = 0.0059) remained independent predictors of sarcopenic obesity in patients with cirrhosis (Table 2). Notably, the inverse association between obese (≥28 kg/m^2^) and sarcopenic obesity (OR 0.076) appears counter-intuitive and may reflect complex interactions between overall adiposity and visceral fat in cirrhosis, which warrants further investigation in the discussion.

**Table 2:** Univariable and multivariable analysis of risk factors for sarcopenic obesity in patients with cirrhosis.

Variables	Univariate		Multivariate	
	OR (95%CI)	*P*	OR (95%CI)	*P*
Age (years)				
<60	ref			
≥60	2.069 (1.406–3.045)	0.0002	1.813 (1.210–2.718)	0.0040
Gender, *n* (%)				
Male	ref			
Female	1.384 (0.945–2.025)	0.0947		
BMI (kg/m^2^), *n* (%)				
<24	ref			
24–28	0.673 (0.442–1.024)	0.0286	0.650 (0.422–1.001)	0.0352
≥28	0.077 (0.019–0.319)	0.0011	0.076 (0.018–0.317)	0.0011
Etiology of liver diseases, *n* (%)				
Viral	ref			
Alcoholic	1.967 (1.200–3.224)	0.0300	1.685 (1.078–2.634)	0.0220
Others	1.584 (0.987–2.540)	0.3292		
Multiple	1.055 (0.501–2.221)	0.3591		
Co-morbidities, *n* (%)				
Hypertension	1.898 (1.278–2.817)	0.0015	1.801 (1.184–2.739)	0.0059
Diabetes	1.577 (1.032–2.409)	0.0352		
Complications, *n* (%)				
Ascites	0.863 (0.591–1.260)	0.4442		
Spontaneous bacterial peritonitis	1.502 (0.867–2.603)	0.1467		
Variceal bleeding	1.703 (0.781–3.712)	0.1807		
Hepatic encephalopathy	1.019 (0.582–1.783)	0.9483		
Decompensated cirrhosis, *n* (%)	0.836 (0.573–1.221)	0.3545		
MELD score	1.000 (0.963–1.039)	0.9870		

BMI, body mass index; MELD, model for end-stage liver disease.

### 3.3 ABAH nomograph by predictors of sarcopenic obesity

**Figure 1:**
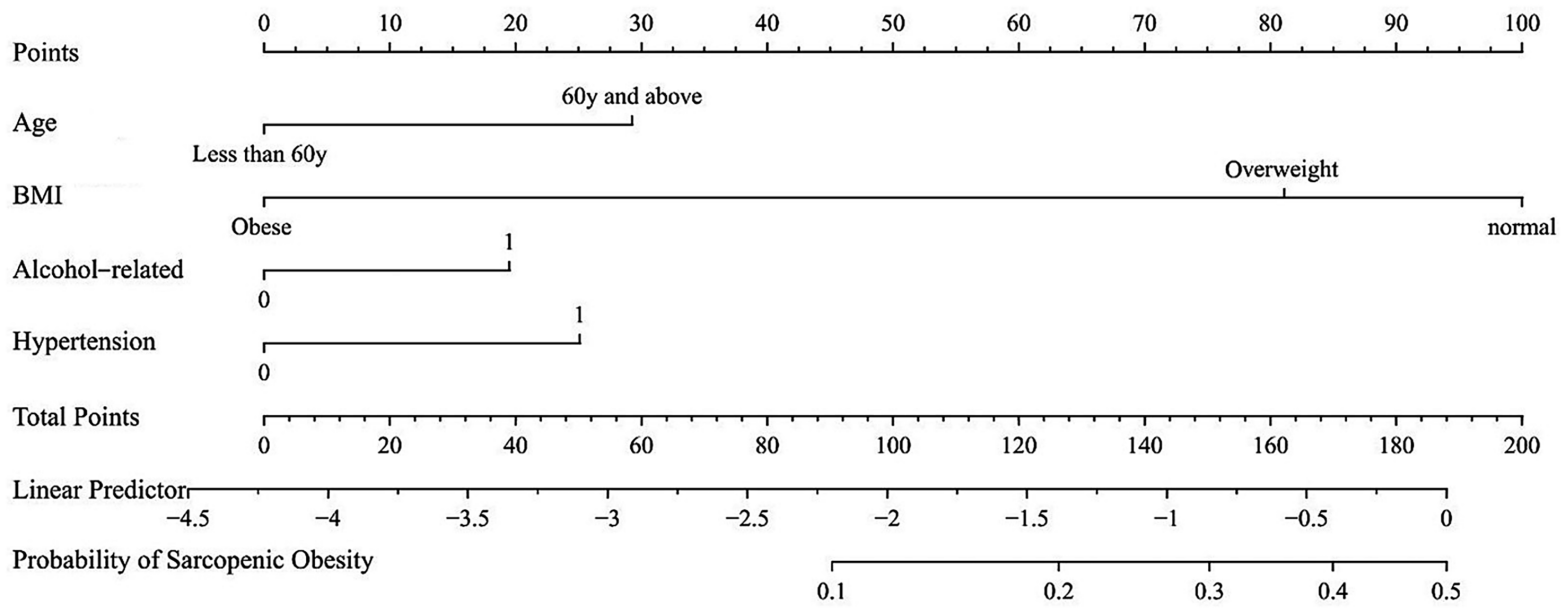
ABAH nomograph based on predictors of sarcopenic obesity. BMI, body mass index; ABAH, age–BMI–alcohol–hypertension.

**Figure 2:**
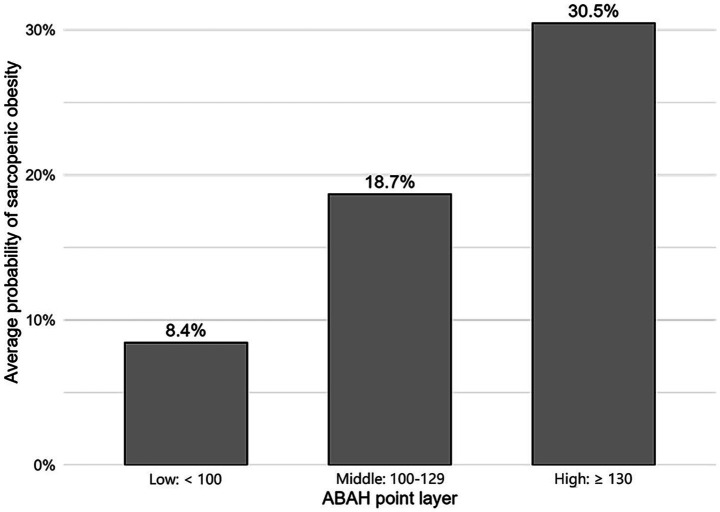
Average risk of sarcopenic obesity by ABAH score. The illustrated group shows an increasing trend, with a *P* for trend = 0.0260. Stratification by ABAH: <100, low; 100–129, middle; and ≥130, high. ABAH, age–BMI–alcohol–hypertension.

A nomograph was developed to integrate age, BMI, alcohol consumption, and hypertension, which were identified as predictors of sarcopenic obesity using multivariate analysis. Each predictor was assigned a score based on its significance. The total points, termed ABAH, corresponded vertically to the linear predictor to estimate the probability of sarcopenic obesity (Figure 1). ABAH scores were categorized into low (<100), medium (100–129), and high (≥130) levels to estimate the average risk of sarcopenic obesity for further subgroup analysis and outcome assessment. The average probabilities were 8.4, 18.7, and 30.5% for the low, medium, and high groups, respectively, showing an increasing trend (P for trend = 0.0260) (Figure 2).

### 3.4 HR of death in patients with cirrhosis by ABAH subgroups

**Figure 3:**
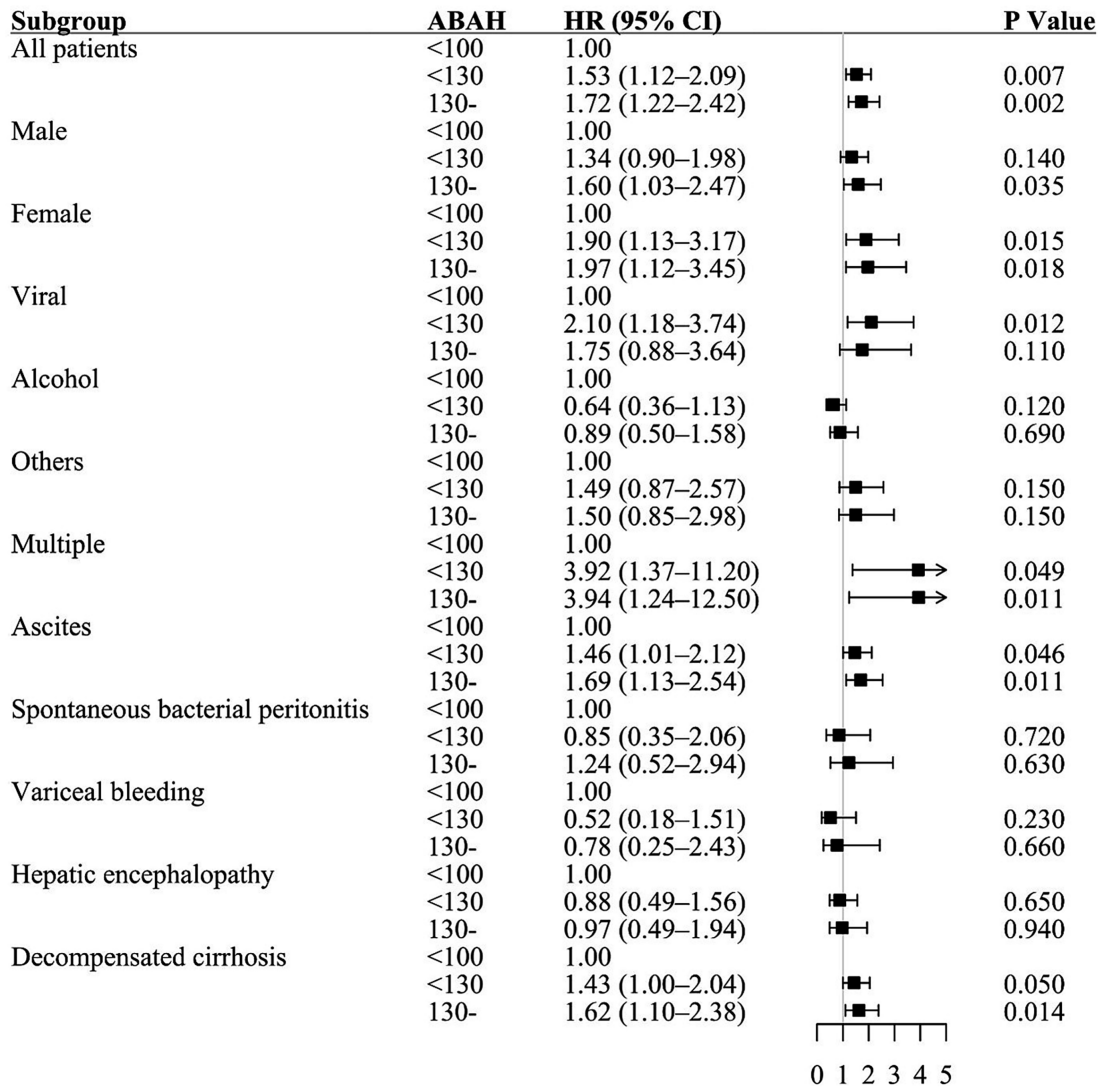
Hazard ratio of death by ABAH score in subgroups of cirrhosis patients. ABAH, age–BMI–alcohol–hypertension.

Among all included patients, the ABAH score (medium vs. low: HR 1.53, 95% CI 1.12–2.09, *p* = 0.007; high vs. low: HR 1.72, 95% CI 1.22–2.42, *p* = 0.002) had significant predictive value for elevated all-cause mortality. A subgroup analysis was conducted to assess mortality risk using the ABAH score. In females, both medium ABAH (HR 1.90, 95% CI 1.13–3.17, *p* = 0.015) and high ABAH (HR 1.97, 95% CI 1.12–3.45, *p* = 0.018) significantly increased mortality compared to that seen with the low ABAH. In males, only high ABAH (HR 1.60, 95% CI 1.03–2.47, *p* = 0.035) showed a significant effect. Similarly, in the viral hepatitis subgroup, high ABAH (HR 2.10, 95% CI 1.18–3.74, *p* = 0.012) predicted increased mortality. Other high-risk subgroups included patients with multiple liver disease etiologies (medium vs. low: HR 3.92, 95% CI 1.37–11.20, *p* = 0.049; high vs. low: HR 3.94, 95% CI 1.24–12.50, *p* = 0.011), ascites (medium vs. low: HR 1.46, 95% CI 1.01–2.12, *p* = 0.046; high vs. low: HR 1.69, 95% CI 1.13–2.54, *p* = 0.011), and decompensated cirrhosis (medium vs. low: HR 1.43, 95% CI 1.00–2.04, *p* = 0.050; high vs. low ABAH: HR 1.62, 95% CI 1.10–2.38, *p* = 0.014). The inclusion of non-significant subgroups further optimized the generalizability of the model (Figure 3).

## 4 Discussion

This study addressed the critical gap in large-scale investigations of sarcopenic obesity in patients with cirrhosis, revealing a 36.5% prevalence of sarcopenic obesity in this population. To streamline risk stratification of sarcopenic obesity, a novel ABAH score—incorporating easily assessable clinical factors (age, BMI, alcohol etiology, and hypertension)—was developed and validated. This score demonstrated a strong predictive utility for the probability of sarcopenic obesity and was associated with all-cause mortality. It is important to clarify that the ABAH score functions as a composite clinical risk marker rather than a direct diagnostic surrogate for CT-defined body composition. It does not replace imaging-based assessment but may help identify patients who warrant closer nutritional and functional evaluation. Overall, our findings support the prioritization of sarcopenic obesity assessment in routine nutritional evaluations of patients with cirrhosis, emphasizing its integration into clinical care to guide early interventions, improve risk prediction, and ultimately enhance patient outcomes.

In our study, 36.5% of patients with cirrhosis were diagnosed with sarcopenic obesity. An earlier review reported 20–35% prevalence of sarcopenic obesity in patients with cirrhosis, with considerable variation depending on the diagnostic criteria and population ethnicity (14). Another study on Caucasians reported a sarcopenic obesity prevalence of 13%, diagnosed using a combination of sarcopenia (SMI < 50 cm^2^/m^2^ for men and <39 cm^2^/m^2^ for women) and visceral obesity (visceral-to-subcutaneous adipose area ratio ≥1.21 for men and ≥0.48 for women) (15). The use of Japanese consensus–based cut-offs is appropriate for our Asian cohort but highlights a key limitation: ethnicity-specific thresholds may limit the direct generalizability of both the reported sarcopenic obesity prevalence and the performance of the ABAH score in non-Asian populations. Therefore, the ABAH score should be interpreted primarily as a risk enrichment tool within similar demographic settings to help identify high-risk patients, rather than as a universal diagnostic surrogate for CT-defined body composition. External validation in diverse cohorts is necessary to confirm its broader applicability. Despite employing different diagnostic criteria, the prevalence of sarcopenic obesity patients we have determined does not differ substantially from that reported in the existing literature (16). Age, inadequate protein intake, basal metabolic rate, sedentary behavior, and body composition indicators have previously been identified as predictors of sarcopenic obesity (17–19). Additionally, Zou et al. reported that sarcopenic obesity significantly increases the risk of hypertension (20). In patients with cirrhosis, factors such as severe malnutrition, amino acid imbalance, insulin resistance, and dysbiosis are also predictive of sarcopenic obesity (7, 21). In addition to age, our study identified alcoholic liver disease and hypertension as independent predictors of sarcopenic obesity for the first time, representing the novelty of this study. Hypertension may serve as a specific predictor of sarcopenic obesity in patients with cirrhosis who do not exhibit hypertension as a prominent symptom. Although alcoholic etiology predicts sarcopenia (22–24), no research has previously reported associations between alcoholic etiology and sarcopenic obesity in patients with cirrhosis.

An unexpected inverse association was observed between BMI and the probability of sarcopenic obesity among patients with cirrhosis. This finding contrasts with conventional assumptions that higher BMI reflects greater risk of sarcopenic obesity. Several mechanisms may account for this discrepancy. First, BMI is an imperfect surrogate for nutritional and body composition status in cirrhosis. It cannot distinguish lean mass from fat mass or capture differences between visceral and subcutaneous adipose tissue, meaning that patients with a “normal” or even low BMI may still demonstrate marked visceral adiposity combined with reduced muscle mass (25). Second, ascites and fluid overload frequently elevate body weight independent of adiposity, distorting BMI. In this context, individuals classified as “high BMI” may in fact be fluid-overloaded rather than obese, while those with relatively little ascites present with lower BMI despite high visceral fat and severe muscle wasting. Third, reverse causation must be considered. Patients who develop sarcopenic obesity may undergo significant involuntary weight loss prior to evaluation, characterized by preferential loss of muscle and subcutaneous fat while maintaining visceral fat; a single time-point CT cannot capture these trajectories, making lower BMI a potential consequence rather than a cause of sarcopenic obesity (25). Finally, differences in adipose tissue distribution rather than global adiposity appear to be the primary driver of sarcopenic obesity in cirrhosis. This finding underscores that within the ABAH score, BMI is not used as a direct proxy for adiposity but rather as a clinical variable that captures the complex limitations of weight-based assessment in cirrhosis, where factors like fluid overload can distort its interpretation. Although CT-based body composition analysis seems to offer superior prognostic prediction, this ABAH score (incorporating BMI) may reduce clinical misjudgements of patient condition based on BMI.

Sarcopenic obesity is also significantly associated with increased mortality risk in older adults and populations with cancer (26, 27). A meta-analysis revealed a markedly elevated all-cause mortality rate in older individuals with sarcopenic obesity compared to healthy controls (28). Notably, compared with older individuals presenting with either sarcopenia or obesity alone, those with sarcopenic obesity had the highest mortality risk (28). Moreover, a large-scale, population-based cohort study identified sarcopenic obesity as an independent predictor of all-cause mortality (27). Similar findings have been reported in patients with liver cirrhosis, where sarcopenic obesity has been linked to higher mortality and identified as a prognostic indicator of overall survival (8, 9). Although our study did not establish sarcopenic obesity as an independent predictor of all-cause mortality in patients with cirrhosis, the current absence of a standardized diagnostic criterion for sarcopenic obesity led us to develop a simple risk factor-based scoring system. The ABAH score demonstrated predictive value for all-cause mortality in this patient population. Its simplicity and ease of use facilitate potential integration into routine clinical management of cirrhosis patients. This risk was particularly pronounced in subgroups with decompensated cirrhosis and ascites, suggesting that sarcopenic obesity may exacerbate portal hypertension and fluid retention, further contributing to poor outcomes.

Despite increasing recognition of sarcopenic obesity in cirrhosis, few studies have attempted to stratify mortality risk using clinical tools. Our ABAH scoring system represents a simple and effective model for estimating the likelihood of sarcopenic obesity and its prognostic implications. Patients were stratified into three risk categories according to their ABAH scores (<100, 100–129, and ≥130), which corresponded to progressively increasing average risks of sarcopenic obesity and associated mortality (Figure 2). This stratification revealed a graded relationship between the ABAH score and HR for sarcopenic obesity, supporting the clinical utility of our model for individualized risk assessment. For example, when integrating it into nutritional assessments, a high ABAH score might trigger early interventions such as personalized nutrition plans, physical therapy, and close monitoring for complications, thereby improving stratified management. Importantly, the predictive performance of the ABAH score remained consistent across multiple clinically relevant subgroups, including female patients and those with mixed etiologies, ascites, and decompensated cirrhosis. These subgroup findings validated the robustness of the ABAH model and highlighted the heterogeneous impact of sarcopenic obesity across different patient populations.

This large-scale study of sarcopenic obesity in patients with cirrhosis developed the novel ABAH score. Alcoholic liver disease and hypertension were identified as predictors of sarcopenic obesity in this population. However, this study has several limitations. First, the retrospective design and single-center setting may have introduced selection bias and limited the generalizability of our findings. Second, the lack of external validation means the ABAH score’s performance needs testing in independent cohorts. Third, using a single time-point CT scan cannot account for changes in body composition over time. Finally, the diagnostic criteria for sarcopenic obesity used in this study—based on L3-level SMI and VATA thresholds recommended by the Japanese expert consensus—may not be universally applicable, emphasizing the need for ethnicity-specific adjustments in future studies. The lack of an internationally standardized definition of sarcopenic obesity, specifically in patients with cirrhosis, remains a challenge and may hinder comparisons across studies. Future prospective, multicenter studies employing standardized definitions and comprehensive outcome data are warranted to validate and extend our findings.

In summary, this study has developed a novel, simple score (age (≥60 years), BMI (normal <24 kg/m^2^, overweight 24–28 kg/m^2^, obese ≥28 kg/m^2^), alcohol-related cirrhosis, and comorbid hypertension) for help predicting sarcopenic obesity and mortality risk in cirrhosis, but its generalizability may be limited by the retrospective, single-center design and the specific diagnostic criteria used.

## Data Availability

The raw data supporting the conclusions of this article will be made available by the authors, without undue reservation.
